# Immunogenic sonodynamic therapy for inducing immunogenic cell death and activating antitumor immunity

**DOI:** 10.3389/fonc.2023.1167105

**Published:** 2023-04-24

**Authors:** Ting Wang, Wangrui Peng, Meng Du, Zhiyi Chen

**Affiliations:** ^1^ The First Affiliated Hospital, Medical Imaging Centre, Hengyang Medical School, University of South China, Hengyang, Hunan, China; ^2^ Institute of Medical Imaging, Hengyang Medical School, University of South China, Hengyang, China; ^3^ The Seventh Affiliated Hospital, Hunan Veterans Administration Hospital, Hengyang Medical School, University of South China, Changsha, Hunan, China

**Keywords:** sonodynamic therapy (SDT), immunogenic cell death, cancer immunotherapy, nanoplatforms, tumor therapy

## Abstract

Immunotherapy is widely regarded as a promising treatment for cancer. However, the immune effector phase suppression of tumor microenvironment (TME) and the generation of immune-related adverse events limit its application. Research indicates that sonodynamic therapy (SDT) can effectively activate antitumor immunity while killing tumor cells. SDT produces cytotoxic substances of tumors, and then cell apoptosis and immunogenic death occur by selectively activating the sonosensitizer under ultrasound. In recent years, various SDT alone as well as SDT in combination with other therapies have been developed to induce immunogenic cell death (ICD) and enhance immunotherapy. This paper overviews the research progress of SDT and nanotechnology in recent years, including the strategies involving SDT alone, SDT-based synergistic induction of antitumor immunity, and immunotherapy based on SDT for multimodal immunotherapy. Finally, the prospects and challenges of these SDT-based therapies in cancer immunotherapy are discussed.

## 1 Introduction

In recent years, immunotherapy, as a new generation of anticancer therapy, has developed rapidly in clinical application, especially checkpoint blockade immunotherapy and chimeric antigen receptor T-cell immunotherapy, which has led to the significant development of clinical research direction (1, 2). Unlike traditional treatment methods, immunotherapy adjusts and strengthens the antitumor effect and produces a therapeutic effect to mobilize the body’s immune function. Under physiological conditions, the immune system can recognize tumor antigens and attack tumor cells with the help of immune adjuvants. However, with the tumor progressed and an immunosuppressive tumor microenvironment (TME) established, immunotherapy failed to drive an efficient immune response (3). As a result, only a few patients have proven to respond to immunotherapy, and adverse immune-related adverse events are often triggered during treatment. Therefore, enhancing the immune reactivity of tumor sites is of great significance for enhancing antitumor immunity.

Induction of local immunogenic cell death (ICD) in tumor areas can transform low immunogenicity into high immunogenicity, which is an effective strategy to potentiate antitumor immunity. After some physical or chemical stimulation, tumor cells can transform low immunogenicity into high immunogenicity, which is an effective strategy to potentiate antitumor immunity (4–6). The host immune response can be reactivated by stimulating the antitumor immune effect, resulting in a better therapeutic effect and prognosis, which is of great significance to improving the prognosis and prolonging the survival of patients.

At present, more and more research has proven that antitumor immunity can be triggered under multiple treatments, such as chemotherapy, radiotherapy, photodynamic therapy (PDT), and sonodynamic therapy (SDT) (7–10). However, chemotherapy and radiotherapy inevitably cause damage to normal tissues, and the phototoxicity and low penetrability of PDT limit its further application. SDT is an emerging cancer treatment based on PDT (11). Similar to PDT, SDT can also be used as an effective cancer vaccine for antitumor therapy (12–14). SDT is a safe and noninvasive local treatment that can selectively kill tumor cells under ultrasound irradiation and cause minor damage to adjacent normal tissues (15–17). The penetration depth in soft tissues can reach tens of centimeters (18, 19), and has excellent potential for inducing immunogenicity and activating antitumor immunity in deep tumor therapy (20, 21).

**Figure 1:**
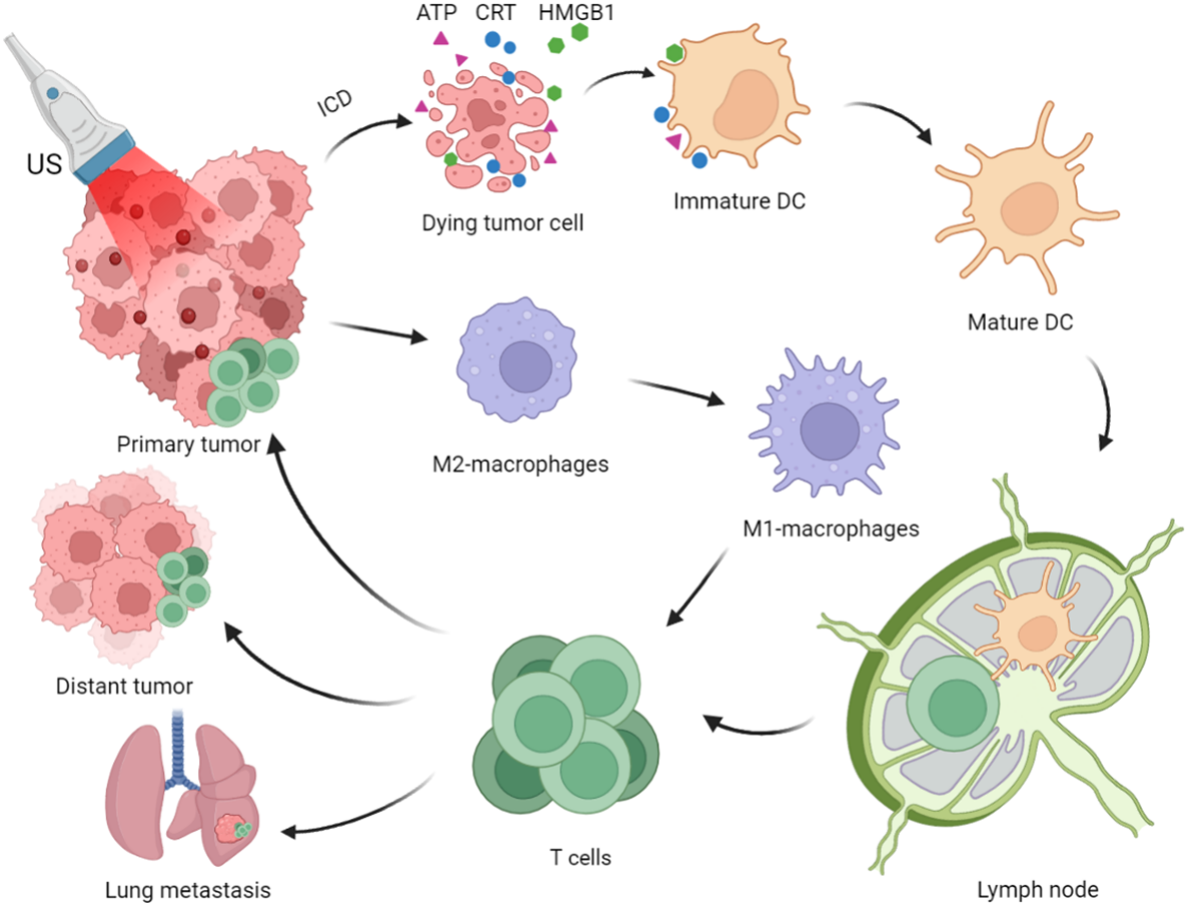
Process of Immunogenic Sonodynamic Therapy in tumor immunotherapy (Created with BioRender.com).

SDT effectively induces and releases the tumor-associated antigens (TAAs) and damage-associated molecular patterns (DAMPs), thereby activating inflammatory responses in TME and draining lymph nodes (dLNs), inducing systemic antitumor immunity and immune memory, and inhibiting tumor growth and recurrence (**Figure 1**) (22, 23). However, cancer immunotherapy based on SDT is insufficient to achieve satisfactory therapeutic effects. Therefore, designing an effective combined treatment strategy for SDT-driven immunotherapy is necessary.

**Figure 2:**
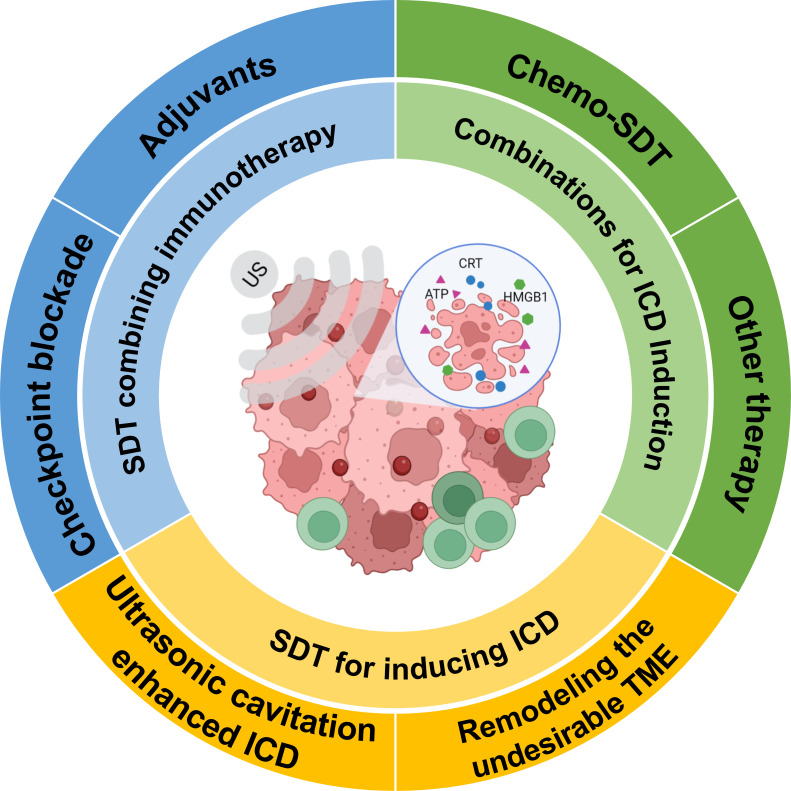
Schematic representation of SDT for inducing immunogenic cell death and potentiating cancer immunotherapy (Created with BioRender.com).

SDT is considered a promising strategy for immune cancer treatment. Combining SDT or SDT-based multimodal therapy with immunotherapy plays an essential role in antitumor immunotherapy (24). In this review, we will discuss the mechanisms of SDT-driven immunotherapy, and then provide an overview of the strategies involving SDT and SDT in combination with other therapies for immune therapy (**Figure 2**). Finally, we conclude with a brief overview of the limitations and future of SDT.

## 2 Main mechanism of SDT induction ICD

SDT has a direct killing effect on tumor cells. To date, the potential mechanisms of SDT have not been fully elucidated. Conventional mechanisms have been accredited that reactive oxygen species produced by sonosensitizers and the cavitation effect induced by ultrasound irradiation act mainly during SDT (25). Activate the enriched sonosensitizer in the local disease site under ultrasound, thereby generating reactive oxygen species (ROS). Extreme oxidative properties stimulate biochemical reactions, including reduced intracellular mitochondrial membrane potential, DNA fracture, cytoskeletal contraction, and chromatin condensation, which causes irreversible damage to tumor cells (26). At the same time, the ultrasound-mediated cavitation effect causes the bubbles in the fluid to contract and expand periodically with ultrasound, which can enhance the permeability of the adjacent cell membrane. The generation of sharp shock when the bubbles undergo rupture can cause mechanical damage to the cells (27).

SDT induces tumor cell death, such as apoptosis and necrosis, and promotes the exposure and release of TAAs, enhancing tumor cells’ antigenicity. At the same time, SDT also generates a series of adjuvant-like signaling molecules, namely DAMPs, including calreticulin (CRT) exposed on the cell surface, high mobility group protein 1 (HMGB1) secreted out tumor cells, adenosine triphosphate (ATP) and heat shock proteins (HSP70, HSP90) released by cells. Coordination of TAAs with DAMPs is necessary to recruit and mature antigen-presenting cells such as dendritic cells (DCs). Exposure or release of DAMPs can be recognized by pattern recognition receptors (PRRs) on the DC cell surface, facilitating DCs recruitment and enhancing the uptake of tumor antigens, initiating a series of cytological responses and ultimately activating antitumor immune responses.

During traditional antitumor treatment, tumor cell apoptosis causes intracellular components to become hypoimmunogenic by activating apoptotic executioner caspase-3 to prevent autoimmunity. Meanwhile, DAMPs exposed to the microenvironment are subject to oxidative degradation and thus lose immunogenicity. Interestingly, partial cell death became more immunogenic during this treatment process by affecting necrosis or necrosis-like cell death (28). Necrosis is considered an inherently immunogenic form of cell death. The disintegration of the plasma membrane induced inflammatory response and antitumor immunoreaction. SDT has the potential to activate cell immunogenic death events. Due to DAMPs released without exposure to harsh conditions that lead to oxidation and proteolysis, the immunogenicity of DAMPs will not be significantly affected.

Hydrophobic sonosensitizers generally preferentially congregate in the hydrophobic inner layers of the plasma membrane, nucleus, endoplasmic reticulum, or mitochondrial membrane. During the ultrasound, the activated sonosensitizer degrades the plasma membrane by lipid peroxidation, thereby leaking intact or less denatured DAMPs to reverse hypoimmunogenicity. Notably, ROS produced can kill tumor cells, trigger endoplasmic reticulum pressure, and damage mitochondria. Endoplasmic reticulum oxidative stress can promote the high expression of CRT and HSPs. The destruction of mitochondria can promote the secretion of ATP and HMGB1. ICD outcomes have generally been accepted to correlate positively with ROS levels. Besides, the cavitation effect of ultrasound can also cause cell membrane cleavage and release of immunogenic DAMPs. Interestingly, it has been proved that ultrasonic cavitation can enhance the rate of ROS production and ICD induction (29, 30). In recent years, several studies related to SDT have demonstrated that SDT induces immunogenic death of tumor cells as an “*in situ* vaccine” that activates the body’s immune response against tumors and has been verified *in vitro* and *in vivo* (31–33).

## 3 Strategies

### 3.1 SDT for ICD induction

Several studies have confirmed that SDT can transform the non-immunogenic “cold” TME into a “hot” TME under the effect of ultrasound, which helps to enhance the effect of antitumor immune response (**Table 1**) (53, 54).

**Table 1:** Main strategy and characterizations of the sonodynamic therapy.

Therapy	Nanoparticles	Characterizations	Model	Refs.
SDT	HiPorfin (HPD)	Immunogenic sonodynamic therapy	Hep3b/H22/S180	(14)
	FA-MnPs	Stronger penetration ability of ultrasound	4T1	(21)
	PFP@PEG-CMD-Ce6 (NBs)	Ultrasound-triggered inertial cavitation (UIC)	CT26	(33)
	LIP-PFH	Ultrasound-triggered inertial cavitation (UIC)	4T1	(34)
	MON-PpIX-LA-CO_2_	Ultrasound-triggered inertial cavitation (UIC)	4T1	(35)
	LMWHA-MPB	Catalyzing H_2_O_2_ to produce oxygen	4T1	(30)
	HABT-C@HA	Catalyzing H_2_O_2_ to produce oxygen	4T1	(36)
	PALF	Reducing oxygen consumption	4T1	(37)
	Mn-MOF	Relieve tumor hypoxia and decrease GSH	4T1	(38)
SDT+Chemotherapy	DTX/X-NPs	Delivery oxygen enhance immunity	B16F10	(39)
	CS–Rh–PFC	Delivering O_2_ to tumor sites	B16F10	(40)
	Lipo-Ce6/TPZ@M_H_	Tumor microenvironment response	B16F10	(20)
SDT+CDT	PEGylated CoFe_2_O_4_ nanoplatforms (CFP)	Catalyzing H_2_O_2_ to produce oxygen	4T1	(41)
SDT+Gas therapy	PIH-NO	Delivering O_2_ to tumor sites	4T1	(42)
	N@CAu-BMSNs	Enhanced tumor-targeting ability	4T1	(43)
SDT+PTT	ZrO_2_-x@PEG/cRGD (ZPR)	Photothermal-augmented SDT	4T1	(44)
SDT+PDT	PARN	Difunctional sono-/photo-sensitizers	B16/Hela	(45)
SDT+PDT+PTT	g-C_3_N_4_/Ce6	Difunctional sono-/photo-sensitizers	4T1	(46)
SDT+PDT+chemotherapy	OIX_NPs	Delivering O_2_ to tumor sites	ID8	(47)
SDT+Immunotherapy	TiO_2_-Ce6-CpG+aPD-L1	Combination with adjuvants	Hepa1-6	(48)
	HMME/R837@Lip+aPD-L1	Combination with adjuvants and checkpoint blockade	4T1/CT26	(49)
	PEG-CDMaPD-L1/Ce6	Combination with checkpoint blockade	B16F10	(50)
	PFCE@THPP*_pf_*-COPs+antiCD47	Combination with checkpoint blockade	CT26	(51)
	SCN@B16F10M/PEG-aPD-L1	Combination with checkpoint blockade	B16F10	(52)

#### 3.1.1 Ultrasonic cavitation enhanced ICD

However, the efficiency of SDT-induced ICD generation remains a limitation. During apoptosis, the immunogenicity of TAAs and DAMPs is inhibited by protein kinase 3 (RIPK3). To improve the immunogenicity of SDT-triggered cell death, Parkp et al. (33) prepared a phase-change nano-sonosensitizer PFP@PEG-CMD-Ce6 (NBs) complex, which was able to cause tumor cell necrosis through bubble mediated cell membrane rupture, but not trigger RIPK3-dependent necrotizing apoptotic through the process of SDT. The expression of HMGB1 in cells was analyzed by Western blot and flow cytometry *in vitro*. The results showed that cancer cells treated with NBs released biologically active DAMPs compared to NPs. The results suggest that cell death induced by ultrasonic cavitation is more immunogenic. Similarly, Yuan et al. (34) constructed LIP-PFH phase-change nanoparticle-mediated SDT to enhance the antitumor immune response by inducing ICD in breast cancer. These results suggest that ultrasonic cavitation-induced cell death is more immunogenic. Further, to amplify the cavitation-enhanced ICD effect, Zhang et al. (29) constructed a nano-sonosensitizer (MON-PpIX-LA-CO_2_) with a continuous cavitation function. L-arginine (LA) has a good function of adsorption/desorption of CO_2_. These complexes can continuously release CO_2_ and induce ultrasound-triggered inertial cavitation (UIC) under ultrasound irradiation. That is conducive to producing abundant ROS, thus successfully inducing robust ICD, more antigen exposure, and presentation enhanced DCs maturation and more activated effector CD8^+^ T cell infiltration *in vitro*. The results suggest that this strategy of ultrasonic cavitation-enhanced SDT-induced ICD successfully converts the “cold” TME into a “hot” one with significantly enhanced suppressive effects in primary and metastatic tumors.

#### 3.1.2 Remodeling the undesirable TME

The TME, such as pH, glutathione (GSH), growth factors, oxygen levels, and immune cells, are closely related to the effect of SDT-induced antitumor therapy. Overcoming the hypoxic microenvironment is necessary to enhance the SDT immune response (35, 55). The breakdown of endogenous H_2_O_2_ to O_2_ using H_2_O_2_ catalysts has been recognized as an effective strategy to alleviate tumor hypoxia and improve the efficacy of cancer therapy. Zhang et al. (56) constructed an *in situ* microenvironmental nano-regulator that can act as an *in situ* oxygen generator and macrophage transducer. LMWHA-MPB has excellent peroxidase activity and generates O_2_ to alleviate tumor hypoxia through the catalytic breakdown of endogenous hydrogen peroxide (H_2_O_2_). In addition, LMWHA-MPB can remodel the phenotype of tumor-associated macrophages (TAMs) after being taken up by M2 macrophages (pro-tumor M2 → antitumor M1). Improving the TME inhibited 4T1 tumor proliferation and metastasis, effectively. Liu et al. (36) constructed a cascade enzyme-based platform (HABT-C@HA) to regulate hypoxia and immunosuppressive factors in TME. The excellent enzyme cascade reaction of HABT-C@HA was utilized to achieve continuous O_2_ production and abundant ROS generation, effectively overcoming hypoxic TME at tumor sites and enhancing the therapeutic effect. The RNA-seq results revealed that HABT-C@HA+US activated immune response and down-regulated MPP2, BHLHE40, and other negative related factors, which improved immune infiltration and reversed breast tumor immunosuppression.

In contrast, reducing oxygen consumption in tumor cells is also a strategy to alleviate tumor hypoxia. Dai et al. (37) constructed a metallic-phenol network-based nano-complex embedded with lactate oxidase (LOX) and atovaquone (ATO), a mitochondrial respiration inhibitor. The nano-complex reversed the tumor’s immunosuppressive state by inhibiting mitochondrial respiration and assisting the lactate depletion process for alleviating tumor hypoxia and acidic TME. It exhibited effective immunostimulatory properties under US irradiation, such as releasing inflammatory factors (i.e., TNF-α, IL-6, IL-12), decreasing polarization of M2 macrophages, and increasing infiltration of activated T cells into tumor tissue, achieving a characteristic enhancement of SDT and inhibiting tumor proliferation and metastasis.

In addition to hypoxia, SDT is also severely limited by high glutathione (GSH) in TME. To improve the efficacy of SDT-induced antitumor immune response, Gan et al. (38) constructed a manganese porphyrin-based metal-organic framework. Mn-MOF exhibited peroxidase-like and GSH-lowering activities *in vitro*. Upon effective internalization into cancer cells, Mn-MOF catalyzed the generation of O_2_ from tumor-overexpressed H_2_O_2_ to alleviate tumor hypoxia. Meanwhile, Mn-MOF reduced intracellular GSH content and GPX4 activity. In addition, Mn-MOF reduced the number of bone marrow-derived suppressor cells in tumor tissues by increasing the number of activated CD8^+^ T cells and mature dendritic cells. Thus, research suggests that it has strong anticancer and antimetastatic activities in the *in vivo* treatment of H22 and 4T1 tumor-bearing mouse models.

### 3.2 SDT-based synergistic induction ICD

Although SDT is widely used for anticancer immunity, it still has some limitations, which are insufficient to elicit a robust immune response. Recently, multiple combination therapy strategies have been used to improve the efficiency of SDT. SDT-based synergistic induction of anticancer immunity is a potential strategy.

#### 3.2.1 SDT combined with chemotherapy for ICD induction

Studies proved that chemotherapeutic drugs, including doxorubicin, oxaliplatin, cyclophosphamide, and paclitaxel, can also promote ICD in tumor cells and elicit host immune responses (53, 54). Chemo-SDT synergistic has produced a more excellent antitumor immune response than SDT alone. For instance, Zhai et al. (39) created a multi-responsive drug release nanoplatform (DTX/X-NPs) that enabled the release of the docetaxel DTX loaded with the cross-linked sonosensitizer chlorin e6 (Ce6) *via* redox/enzyme/ultrasound responsive for combined chemo-sonodynamic to initiate antitumor immune responses. Cytotoxic lymphocyte (CTL) infiltration increased in the TME following Chemo-SDT compared to the CTL percentage in the SDT group, and the CTL percentage increased by 1.3%. In addition, both SDT-NPs and Chemo-SDT treatment increased INF- expression, with a more pronounced treatment trend for Chemo-SDT. The aforementioned experimental findings show that chemo-SDT improves immune activation and the effectiveness of fighting *in-situ* cancers vs. metastasis.

The relationship between hypoxic tumor tissue and sustained oxygen depletion severely hampers the antitumor effect of oxygen-dependent Chemo-SDT. To enhance the Chemo-SDT antitumor immune response, Zhai et al. (40) designed a novel redox/ultrasound-responsive oxygen-carrying nanoplatform (CS–Rh–PFC). The CS–Rh–PFC encapsulated sonosensitizer Rhein (Rh), chemotherapeutic medication docetaxel (DTX) and perfluorocarbon. PFC transports oxygen and raises the oxygen concentration of B16F10 melanoma cells, finally enhancing the effectiveness of Chemo-SDT-induced ICD. Notably, DTX-loaded CS-Rh-PFC NPs elicited more “eat-me” signals and had higher CRT exposure on B16F10 cells. Increased secretion of IFN-γ, TNF-α, IL-2, and IL-6 cytokines and increased levels of CD4^+^ and CD8^+^ T cells infiltrating the tumor after treatment suggested that immunogenic chemotherapy-ultrasound kinetic treatment based on oxygen-carrying nanoparticles could significantly activate the immune system.

Utilizing the particular hypoxic tumor environment for SDT combined with hypoxia-induced chemotherapy is also an effective strategy. Wang et al. (20) constructed a biomimetic decoy, loaded the sonosensitizer Ce6 and hypoxia-activated tirapazamine (TPZ) in pH-sensitive liposomes, and fused them with PLT and RBC membranes to produce lipid Ce6/TPZ@M_H_. Ce6 generates toxic ROS upon US irradiation, and the resulting hypoxia microenvironment activates TPZ for high-effective synergistic therapy. SDT combined with hypoxia-induced chemotherapy induces ICD synergistically, releasing the DAMPs (including CRT, HMGB1, etc.) and successfully promoting antitumor immunotherapy. Meanwhile, Lipo-Ce6/TPZ@M_H_ decoys maintain binding interactions with high levels of HMGB1 to prevent platelet-mediated tumor metastasis. Combined treatment with SDT and hypoxia-activated TPZ shows excellent potential in eliminating tumors *in situ* and inhibiting lung metastasis from melanoma.

#### 3.2.2 SDT combined with other therapy for ICD induction

In addition, several other combination therapy modalities have been explored for synergistic induction of ICD, such as gas therapy, photothermal therapy (PTT), PDT, chemodynamic therapy (CDT), etc. Gas therapy delivers gases, e.g., carbon monoxide (CO) and nitric oxide (NO), to tumor sites to relieve and treat disease. Liu et al. (43) developed an ultrasound-driven biomimetic nanosystem (N@CAu-BMSNs) to verify whether SDT/CO gas therapy could trigger burst ICD. In this study, the expression of CRT, as a biomarker during ICD, in 4T1 cells after N@CAu-BMSN treatment was detected. Compared to the control group, N@cau-BMSN+US-treated 4T1 tumor cells could more effectively increase CRT expression *in vitro* and *in vivo*. In addition, effective immune response and long-term immune memory were achieved by combining with indoleamine 2,3-dioxygenase (IDO) signaling blockade. Based on SDT/Gas therapy and IDO signaling inhibition may be promising strategies to prevent tumor recurrence and lung metastasis in future clinical translation. Ji et al. (42) designed a US-responsive oxygen and NO-loaded sonosensitive nanoparticle (PIH-NO) for combined SDT/NO gas therapy. The effectiveness of sensitization was validated on the breast cancer model *in vitro* and *in vivo*. PIH-NO preferentially accumulates in mitochondria, and the burst release of O_2_ and NO under US treatment simultaneously generates large amounts of ROS and RNS, enhances SDT to inhibit tumor growth, and amplifies ICD. Furthermore, PIH-NO promoted the maturation of DCs and caused the clustering of M2 macrophages to M1 phenotype, reduced MDSC recruitment, reversed immunosuppression of TME *in vivo*, and enhanced immune response. Studies have demonstrated that O_2_-enhanced SDT combined with NO treatment induces and amplifies ICD, triggering an antitumor immune response.

Most photosensitizers, such as Rose Bengal (RB), Ce6, and indocyanine green (ICG), are also sensitive to ultrasound. Liu et al. (45) designed a nano-sonosensitizer (PARN) consisting of difunctional sono-/photo-sensitizers (RB) for SDT combined with PDT, which has a good immune activating antitumor effect and a favorable prognosis. Chen et al. (46) developed a metal-free g-C_3_N_4_/Ce_6_ nanohybrid.Metal-free g-C_3_N_4_ nanosheets loaded with Ce6 as a dual-function photo/sonosensitizer. Under ultrasound and NIR irradiation, the g-C3N4/Ce6 nanoplatform significantly combines PDT and SDT with pronounced antitumor effects. More importantly, the photothermal greatly promotes immunoreaction, significantly enhancing long-term immune responses and inhibiting tumor recurrence in 4T1 tumor-bearing mice. Chang et al. (47) prepared phase-changeable core-shell nanoparticles (OIX_NPs) with an oxygen-carrying core and the photosensitizer indocyanine green (ICG)/oxaliplatin (OXP) in the shell for PSDT (SDT/PDT) combined with chemodynamic therapy for ovarian cancer. This combined strategy can induce ICD through the passive release of HMGB1 and promote surface exposure of CRT. In a bilateral syngeneic mouse model, OIX_NPs mediated PSDT promoted infiltration of cytotoxic T lymphocytes within the tumor, inhibiting the primary tumor and the growth of distant tumors. The study suggests that PSDT combined with chemodynamic therapy is an effective therapeutic strategy to induce systemic antitumor immunity.

Similar to PDT, noninvasive PTT converts light into heat. PTT is based on near-infrared light (NIR-II) absorption-mediated photothermal conversion therapy. It has been shown that mild PTT could alleviate the hypoxic conditions in the tumor region and facilitate SDT-mediated ROS generation (59). Xue et al. (44) developed an oxygen-deficient zirconia-based nanoplatform with surface PEGylation and cyclic-Arg-Gly-Asp (cRGD) peptide functionalization (ZrO_2_-x@PEG/cRGD, ZPR). It successfully induces ICD and promotes the photothermal enhancement of SDT antitumor effects in the NIR-II biological window. Upon confocal microscopy, SDT/PTT enhanced the CRT expression of ZPR+L/US. Compared with the control group, the intracellular CRT level in the ZPR+L/US group increased about 1.86-fold compared with the ZPR+US group, while the HMGB1 release decreased by about 55.7% and the intracellular ATP level decreased by about 60.5%, which was consistent with the extracellular decrease level. Overall, ZPR NPs promoted the ICD more significantly under NIR-II/US irradiation due to the crud-based ligand anchoring effect.

CDT utilizes Fenton or Fenton-like reagents (typically Fe^2+/3+^) to catalyze excess H_2_O_2_ producing high ROS that kill tumor cells and have been shown to trigger ICD (58, 60). Xue et al. (41) synthesized a bioreactor PEGylated CoFe_2_O_4_ (CFP) for augmented SDT/CDT and elicit robust immune response by a typical solvothermal method. CFP is a novel and efficient SDT sonosensitizer with peroxidase-like activity, which can react with endogenous hydrogen peroxide to generate molecular oxygen. High O_2_ levels may promote ^1^O_2_ production during SDT. Besides, the fenton-like reaction can be produced by the Co^2+/3+^ and Fe^2+/3+^ redox pair by CoFe_2_O_4_ to produce ROS for CDT. The therapeutic effect of CFP-mediated SDT/CDT combined with anti-PD-L1 checkpoint blockade was also further evaluated in an aggressive lung metastasis model in BALB/c mice carrying bilateral 4T1 tumors. A few metastatic nodules were found in “CFP+US+aPD-L1” mice. CFP-enhanced SDT/CDT combined therapy effectively triggered ICD and promoted antitumor immunity while suppressing primary and distant tumors.

### 3.3 Multimodal immunotherapeutics on basis of SDT-induce ICD

Various strategies have been explored to enhance SDT and induce ICD in tumor cells to activate the host immune response to cancer. Based on the successful induction of the ICD, cancer immunotherapy can be triggered more effectively by fully activated antigen-presenting cells. However, the immunosuppression-related phenotype of tumor cells can interfere with the recognition of tumors by effector T cells, thus reducing the efficacy of tumor immunotherapy. Therefore, unimodal cancer immunotherapy based on SDT is insufficient for satisfactory treatment. Consequently, it is necessary to use immunotherapy on basis of SDT for multimodal cancer immunotherapy (57, 61, 62).

#### 3.3.1 Combination with checkpoint blockade

Immune checkpoints are a class of immunosuppressive molecules that regulate immune responses, thereby avoiding damage and destruction of normal tissues, which become one of the main causes of immune tolerance during tumorigenesis and development. Immune checkpoint blocking (ICB) is a therapeutic approach to regulate T-cell activity to kill tumor cells through pathways such as co-inhibition or co-stimulatory signaling (63). For example, Chen et al. (51) constructed perfluorocarbon-loaded fluorinated covalent organic polymers (PFCE@THPP*_pf_*-COPs). When injected intratumorally, PFCE@THPP*_pf_*-COPs alleviated tumor hypoxia and inhibited tumor growth by inducing ICD in cancer cells under ultrasound irradiation. Combined anti-CD47 immunotherapy can synergistically inhibit tumor growth and recurrence by increasing the efficiency of tumor infiltration by M1 macrophages and cytotoxic CD3^+^ and CD8^+^ T cells while decreasing the efficiency of immunosuppressive regulatory T cells.

To improve the delivery efficiency of immune checkpoint inhibitors and to reduce adverse immune reactions, Li et al. (52) prepared nano-sonosensitizers loaded with programmed cell death ligand 1 antibody (aPD-L1), and modified malignant melanoma cell membranes (B16F10M) for targeting melanoma. The bionanoparticle SCN@B16F10M/PEG-aPD-L1 was used for homologous and immune checkpoint dual targeting and enhanced sonodynamic tumor immunotherapy. The functionalized nano-sonosensitizers showed visible long-term retention in the tumor, which facilitated synergistic dual targeting of homologous and immune checkpoints and enhanced *in vivo* SDT-immunotherapy. A novel TME-responsive nano-sonosensitizers design strategy has high spatiotemporal specificity in the drug-controlled release. Shuai et al. (50) constructed pH and MMP-2 dual-responsive acoustic sensitizer PEG-CDMaPD-L1/Ce6 with low pH and high MMP-2 expression in the TME to trigger *in situ* release of aPD-L1. This strategy of *in situ* induction of ICD and release of aPD-L1 has better targeted therapeutic effects and can induce strong anticancer immunity and long-term immune memory.

#### 3.3.2 Combination with adjuvants

In addition to SDT combined with immune checkpoint inhibition therapy, immune adjuvants are also used to enhance the antitumor immune response. Cytosinphospguanine (CpG), a toll-like receptor 9 (TLR9) agonist, is a significant antitumor immune adjuvant in clinical research (64). For example, Wang et al. (48) constructed TiO_2_-Ce6-CpG using titanium dioxide (TiO_2_) as a carrier loaded with the sonosensitizer Chlorin e6 (Ce6) and the immune adjuvant CpG oligonucleotide (CpG ODN). The emerging nano-sonosensitizer (TiO2-Ce6-CpG) effectively kills tumor cells and triggers ICD under ultrasound irradiation. The immune adjuvant CpG stimulates the immune system to activate adaptive immune responses. Combined aPD-L1 treatment showed superb inhibition against primary and metastatic tumors in mice’s bilateral subcutaneous model of hepatocellular carcinoma. Besides, Chen et al. (49) designed a nanosonosensitizer, co-encapsulated HMME and immune adjuvant R837 in liposomes (HMME/R837@Lip). It has demonstrated that the nano-sonosensitizer can enhance the effect of SDT through applicating in multiple tumor models. SDT + PD-L1 blockade enhances the suppression of primary and distant tumors in the 4T1 breast cancer and CT26 colorectal cancer models. In addition, this combination therapy strategy provides a long-term immune memory function that can prevent tumor recurrence.

## 4 Discussion

More and more studies have confirmed the application value of SDT in antitumor immunotherapy. However, there are still various challenges in inducing immunotherapy during SDT. SDT-induced immune responses are of limited efficiency. In terms of treatment strategies, targeted delivery of sonosensitizers to specific organelles, such as the endoplasmic reticulum, and mitochondria, is expected to enhance the immunogenicity of tumor cells further. Endoplasmic reticulum (ER) is a crucial location for ROS production and ICD induction during SDT. The sonosensitizers accumulated in the ER significantly impact the activation of ICD (5). However, in most current studies, sonosensitizers are unable to the subcellular localization of ER, and can only produce ER stress response through indirect ROS activity. Therefore, a sononanoplatform that can directly target ER and effectively trigger ER stress is required. Peptides targeting ER, such as pardaxin peptides, and decorated nanomaterials are expected to solve the problem (65). Targeting peptides-modified nanoparticles could carry sonosensitizers accumulated specifically in the ER. The ER-localized SDT strategy improves primary ROS production and provides a promising modality for ICD-assisted immunotherapy (19). In terms of treatment mode, the effect of a single SDT treatment is often limited. Therefore, combining multiple modes is the treatment method to improve the efficiency of antitumor immunity. Many studies have confirmed that different strategies, such as SDT combined with chemotherapy, PDT, PTT, and SDT combined with immunoblockers are expected to improve the effect of tumor immunotherapy.

Although SDT-synergized immunotherapy has rapid development, several limitations associated with SDT and immunotherapy remain to be addressed. Combining immune checkpoint blockade therapy is often administered by systemic injection, leading to insufficient drug targeting, low drug utilization, and even susceptibility to immune-related adverse events. Targeting tumor delivery by loading immunotherapeutic drugs onto nanocarriers can solve these problems. The integration of sonosensitizers with various nanoplatform overcomes the challenges of SDT, such as hypoxia and poor targeting, and enhances SDT-based ICD induction and immunotherapy through synergistic delivery strategies. However, in clinical translations, the stability and biocompatibility of nano-sonosensitizers need to be evaluated.

In addition, even though tumor-derived HMGB1 is critical for SDT-related immunogenicity, researchers found that HMGB1 is also involved in tumor progression. Wang et al. proved that extracellular HMGB1 is an essential factor for TLR4 interaction with platelets and promotes melanoma tumor cells’ interaction with aggregation, extravasation, and metastasis. Preventing HMGB1-mediated tumor growth and metastasis should be further studied (20).

In summary, the antitumor immune response is a highly complex process, and disruption of any of these steps can reduce the effectiveness of antitumor immunotherapy. In the future, it is necessary to design more effective multifunctional sonosensitizer nanoparticles rationally to obtain satisfied antitumor immunotherapy.

## Author contributions

TW (First author): Conceptualization, investigation, writing - original draft. WP: Writing - Original draft. MD; ZC (Corresponding Author): Conceptualization, funding acquisition, resources, supervision, writing - review & editing. All authors contributed to the article and approved the submitted version.
